# Evaluation of a gamification and flipped-classroom program used in teacher training: Perception of learning and outcome

**DOI:** 10.1371/journal.pone.0236083

**Published:** 2020-07-16

**Authors:** Cosme J. Gómez-Carrasco, José Monteagudo-Fernández, Juan R. Moreno-Vera, Marta Sainz-Gómez

**Affiliations:** 1 Department of Mathematics and Social Sciences Teaching, University of Murcia, Murcia, Spain; 2 Department of Evolutionary and Educational Psychology, University of Murcia, Murcia, Spain; Universitá degli Studi di Bergamo, ITALY

## Abstract

Recent years have witnessed the arrival of new methodological horizons in teacher training. Technological resources and mobile connections play a major role in these studies. At the same time, there is a focus on play to increase commitment and motivation. It is in this context that gamification and flipped-classroom strategies have arisen. This paper presents the results of a training program with future Primary Education teachers using gamification and flipped-classroom strategies and techniques. The aim was that teachers in training acquire competences in proposing innovative teaching units. The learning achieved through the program was evaluated by collecting perceptions via a questionnaire and using an observation scale of the didactic units designed. The program was implemented in four classroom groups (N = 210) at the University of Murcia (Spain). Descriptive statistics are shown; mean tests (t of Student and ANOVA of one factor); non-parametric tests (U-Mann Whitney test); and Pearson correlations between subscales. The data show a very positive assessment of the learning achieved and the strategies applied in the training program. The learning outcomes were satisfactory, although lower than perceived. Some differences between class groups and gender are discussed, and some weaknesses of the program are pointed out.

## Introduction

### Gamification and the flipped-classroom in teacher training

Recent years have witnessed the arrival of new methodological horizons in teacher training [1] and international studies have stressed the need to renew teacher training programs to improve teaching-learning processes in compulsory education [2–4] and have emphasized mastery of normal classroom tasks [5, 6]. Technological resources and mobile connections play a major role in these studies [7, 8]. At the same time, there is a focus on play to increase commitment and motivation [9–11]. It is in this context that the phenomenon known as gamification has arisen [12, 13]. Gamification is based on the argument that many traditional activities (including school activities and traditional learning) are not intrinsically interesting and the understanding that the introduction of characteristics similar to games would make them more attractive [14]. One technique in gamification is the introduction of rewards when a user reaches a specific goal [15]. There is a scoring system and a record of achievements, classifications (overall or partial), and users can win badges [9].

Gamification is on the rise in education [16]. The success of gamification strategies lies in increasing students’ motivation [17]. Motivation is one of the most acclaimed potentialities of gamified methodologies, an act that converts this technique in a useful formative strategy in the various stages of education, even between trainee teachers [18].

In this sense, several research papers have analyzed the intrinsic and extrinsic results of the motivation of the students prior to gamification strategies [19, 20], finding that gamification per se does not guarantee greater motivation, but must be focused on achieving learning results, so the type of game must be adapted to the contents to be worked on and to the characteristics of the students who participate in it [21, 22].

Authors such as [23] believe that the use of gamification in Higher Education is positive, as the strategies mean that students are more involved in teaching and learning processes. They become the focus of the teaching, thus fostering motivation [24]. The positive effects in the research have enjoyed empirical support [9, 25] based on key elements like scores, awards or rewards [12, 26, 27].

This change sought through the incorporation of gamification to enhance motivation is closely related to the SAL (Student Approach to Learning) approach. Although this approach has been promoted by the European Union for Higher Education Studies since the Bologna Plan, studies show that teacher training programs still retain some ineffective instructional strategies [28], which has led researchers to propose alternative approaches, such as the flipped-classroom [29]. Thanks to the support of educational technologies, the flipped-classroom has become a viable pedagogical approach that addresses the needs of today’s students [30]. The flipped-classroom allows greater student practical activity in the classroom, leaving the transmission of conceptual knowledge for home video viewing [31]. Like other technological strategies, its success depends on good educational planning, and specifically it should comply with the three levels of the T-PACK (Technology, Pedagogy And Content Knowledge) framework [32–34].

It is still a strategy with relatively little empirical research in academia [35]. Like other teaching actions focused on active student learning, the flipped-classroom improves traditional formulas [36], increases student participation [37] and, at the same time, admits research and critical activity. Studies show that a direct consequence of this methodology is increased motivation and greater experiential learning [26, 38].

Many authors have indicated the necessity of improve the teaching practice in teacher training to train highly-qualified teachers. This is an issue of current interest on an international level [2, 39–41]. The focus of this article is key: the effects of an intervention programme in teacher training on the knowledge, skills and competencies of trainee teachers [42]. There is an extensive bibliography on teacher training with a large number of authors pointing out that more comparative research is necessary and that empirical studies in higher education need to have systematic contact with the subsequent results [43]. Studies in other contexts in Higher Education have shown how active teaching strategies improve student motivation and competencies [35, 44–47]. The purpose of this article is to show the positive effects of these active strategies in teacher training on the improvement of their skills as future teachers (in the ability to design teaching activities). The novelty of this article is the linking of the flipped classroom as a method of daily work in the classroom and gamification as a motivation technique. Research in higher education has usually focused on one of these strategies. In this article we show the positive effects of linking both teaching methods.

Gamification and flipped-classroom has drawn the attention of teachers and academics in the last five years, but there is still an underdeveloped theoretical and empirical frame to discuss the effectiveness of these methods, above all used both together like in this study [48], in spite of we already have the first evidences in primary education [49]. But there is a lack of evidence and empirical evaluation of its use in a non-entertainment context [50] in higher education, where they are less used and there are no many examples of these methods [45]. Above all in Social Studies Education, a field still very linked to traditional teaching & learning methodologies, where it could be found studies in economics and marketing [51], social work [52], business courses [47, 53], educational technology programs [54], employees, consumers or environmental experiences [55] but very few researches of gamification and flipped-classroom teaching History or Geography lessons [56] and never using both strategies at the same time in this field [57–60].

Empirical investigations need to dig into evidences designing Social Science educative programs using gamification and flipped-classroom. It could be very interesting to know what is the opinion of future teachers [61] using these methods together or what are the perceptions of the training teachers about learning outcomes or self-motivation which is especially important for educational research as the students of today will be the teachers of tomorrow [62]. And, in the future, their opinion about strategies and methods will affect their curricular and pedagogical decisions during their professional development [63] which, as well, can influence the future learning of their students [64].

### Research question and aims

The objective of this paper was to analyze the effects of a training program based on flipped-classroom and gamification on the learning perceptions and outcomes of teachers in training.

The specific objectives derived from this general objective are:

SO1: To analyze the opinion of future teachers on the strategies used in the training program by group, gender and flipped-classroom/gamification techniques.

SO2: To analyze the perception that future teachers have of the learning achieved in the training program by group and gender, and relationship with the techniques assessment subscale.

SO3: To analyze the learning outcomes of future teachers in relation to their ability to make teaching proposals for Social Sciences in Primary Education and the differences by groups and learning perceived.

## Method

### Participants

This specific study was reviewed and approved by an institutional review board (Research Ethics Commission of the University of Murcia) before the study began. We obtained the written consent associated to the Ministry of Science, Innovation and Universities investigation project. After informing to the participants about the research objectives, they signed an informed consent document (supplementary material).

The research sample comprised 210 trainee teachers (53 males; 25% and 157 females; 75%), with respect to the groups, in the group 1 (bilingual) is in the one that is smaller proportion of males with respect to the other groups (Table 1). The total number of participants in the sample study the third course on the Primary Education Degree at the University of Murcia, Spain. The purpose of the academic degree is the initial training of Teachers trained to practice in the Primary Education stage (6–12 years). The age range of the participants was 19 to 44 years (M = 20.94 and SD = 2.77). Almost 90% of the sample was between 19 and 22 years old (Table 1), which indicates that the academic year of the majority of students corresponds to the age. The training program was carried out in four classroom groups, although all students belonged to the same course, who are required a high cut-off note to access the Degree (2018/19 academic year—8.506). There are differences between group 1 and the other groups (2, 3 and 4). Group 1 differed from the others because it is a bilingual group (a minimum of 15 subjects are taught in English). In addition, students in the bilingual group have to meet the following requirements: have the nationality of countries whose official or co-official language is English, either, have a certificate of accreditation of a level B1 or higher in English and have completed a Bilingual Baccalaureate. Groups 2, 3 and 4 are more homogeneous both in percentage of men and women and in the academic origin of the participants.

**Table 1:** Sample and socio-demographic information by group.

*a) Distribution of the sample by group and socio-demographic information*										
Group		Frequency		Percentage		Monolingual /Bilingual		Sex of the participants		
								Male		Female
1		44		21,0		Bilingual		7		37
2		54		25,7		Monolingual		14		40
3		60		28,6		Monolingual		13		47
4		52		24,8		Monolingual		19		33
*b) Socio-demographic information (in terms of age) of participants according to groups*										
Ages of the participants										
Age groups	19–21		22–24		25–28		29 and over		Total	
Percentage	86,5		7,7		2,4		3,4		100	

It is interesting to know whether the students of the same obtain benefits in the objectives proposed in this work with respect to their colleagues in the monolingual groups, as well as to check the uniformity in the implementation of the program based on gamification and flipped classroom in teacher training and in the process of teaching and learning through the different groups.

Futhermore, the distribution of the sample was quite homogeneous: 21% in Group 1 to 28.6% in Group 3 (Table 1). In addition, this table presents information about participants' demographics based on groups.

### Research focus

A methodological approach based on program evaluation was chosen: design, implementation and evaluation of a training program [54]. For evaluation of the program, a quantitative approach was applied using two tools: a questionnaire with Likert scale (1–5) to ascertain the perceptions of the participants about their learning; and an observation record of the training units designed by the teachers in training to ascertain the learning outcomes.

### Design of the training program

The training program was run in four class groups on the subject *Teaching Methodology for Social Sciences* on the Primary Education Degree at the University of Murcia (Spain). To ensure fidelity in the implementation by the teaching team, a document was created with a protocol (supplementary material). This document is a checklist of activities that teachers should complete to ensure uniformity in the implementation of the program. The aim of the subject is that the students acquire competences in the design of innovative teaching proposals for social sciences in Primary Education. The strategies used in the training program were based on the flipped-classroom, as a teaching approach, and gamification, as a technique to encourage motivation. The subject was taught in the first semester of the academic year 2018/2019 (September-December), with two sessions of two hours a week. The teaching team produced a weekly video with the theoretical contents of the subject. For the flipped-classroom, the students had to watch the video at home. The activities inside the classroom were based on case studies, simulations, analysis of materials, cooperative work, etc. This was combined with gamification techniques. To design our gamification techniques we follow the claims of Teixes [65] on the different mechanics and dynamics used. Specifically, in terms of dynamics, which are the elements that make progress in the game visible, we used the *points*, specifically experience points. Those are earned from the actions performed by users: the number of successes that the member obtained from the questions launched with the Socrative application. A *classification* was also introduced, that is, an element that visually ordered users according to the score achieved. As for the dynamics, we use a system of *rewards*, that is, a valuable element that is obtained after the achievement of an objective. In our case it was a bonus in the final grade of the subject based on the total points obtained at the end of the experience. Another dynamic was the competition by comparing the results of all groups through the classification, thus seeking extra motivation. Finally, the *feedback* was used: the interrelations that was offered to the students at the end of each questionnaire so that they knew their degree of progress in the gamified system. With this feedback, students were stressed on the need to achieve a real learning of the contents of the subject, leaving the rewards in the background. In this way we try to boost intrinsic motivation against extrinsic.

At the beginning of each of the sessions the students answered questions about the theoretical videos through team competitions made using the Socrative platform, following the recommendations of studies like [66]. At the end of the sessions, team competitions were held again on the contents dealt with throughout the session. The groups could obtain badges during the development of the proposal, and prizes at the end of the course for those who gained most badges related to the final grade.

Throughout the program the working groups had to design an innovative teaching unit for social sciences. At the end of the course, the groups were required to give an oral presentation with a simulation of one of its parts and to carry out the activities designed *in situ*. The unit was evaluated using an observation scale (supplementary material) in order to test the effectiveness in learning competencies related to the design of innovative proposals.

### Tools used for data collection

The information on the effects of this training program was collected through two tools. First, there was an ad hoc questionnaire entitled "Evaluation of the gamification and flipped-classroom based training program", which used a closed Likert rating scale (1–5), consisting of three thematic blocks (supplementary material). The first block addressed the perceptions of trainee teachers on how the program had affected their motivation. The second block of the questionnaire dealt with how satisfied they felt with the program. The third block focused on the perception of the learning received in the training program. For this purpose, a series of statements related to each of the objectives of the program regarding the proposal of innovative teaching units were drawn up. Participants were also asked to assess the role that, in their opinion, each of the strategies and techniques used in the effectiveness of learning played in learning effectiveness.

The design of the questionnaire took into consideration other studies on the effects of gamification programs on motivation, satisfaction and learning effectiveness [9, 19, 67, 68]. The validation of the content was carried out by a panel of experts who judged the relevance and clarity of the items in the tool.

The second tool was an observation scale to evaluate the teaching units designed by the future teachers (supplementary material). It had a 1–5 rating scale and was built around four variables that were evaluated by the teaching team: suitability of the structure of the teaching unit; relevance of the training activities; methodological suitability; correction of the evaluation procedures and instruments. The learning outcomes of each of the assessments (1–5) were detailed. Some models developed and implemented in this area of knowledge in observation scales were taken into account [69–71]. Validation of the content was also carried out through a pane l of experts who judged the relevance and clarity of the tool’s variables and the proposed learning outcomes for each of the assessments with the scale.

### Data analysis procedure

The data collected by the two tools were coded and analyzed separately with SPSS v.22.0 for MAC. The reliability and validity of the construct of the perception of learning questionnaire were estimated prior to the data analysis. The internal consistency method based on Cronbach's Alfa used to estimate the reliability of a measuring instrument composed of a set of items of Likert scale type expected to measure the same theoretical dimension (the same construct) was used to analyze the reliability of the questionnaire. This validation procedure has been used in other history education research [69]. The criterion established and used by various authors is that a Cronbach alpha value between .70 and .90 indicates a good internal consistency for a one-dimensional scale [72, 73]. In the case of the questionnaire, satisfactory results were obtained both on a global scale and on each of the subscales used in this study. The degree of reliability of the global scale was also shown to be adequate using the Guttman split half technique (Table 2).

**Table 2:** Cronbach’s alpha internal consistency coefficients and Guttman split half for the scale “evaluation of the gamification and flipped-classroom based training program” and the sub-scales used in the research.

Scales and sub-scales	Number of Elements	Cronbach’s Alpha	Guttman’s split-half
Overall Scale “Evaluation of the gamification and flipped-classroom based training program”	37	.940	.903
Sub-scale “perception of learning”	8	.876	
Sub-scale “perception of motivation”	13	.821	

The validity of the construct and the viability of a subsequent factorial analysis were also checked. For this purpose, the correlation matrix was analyzed and Barlett's sphericity test and a Principal Component Analysis (PCA) were carried out for each of the blocks of the questionnaire. The exploratory ACP explains the maximum percentage of variance observed in each item from a smaller number of components which summarize that information [74].

The analysis of the correlation matrix looked for variables that did not correlate well with any other, that is, with correlation coefficients of less than 3; and variables that correlated too well with others, that is, variables that have some correlation coefficient greater than 9. In the case of the study questionnaire, no variable with these characteristics was found.

In the three blocks a critical level (Sig.) of .000 was obtained in Barlett's sphericity test. If we apply the ACP to each of the blocks, we obtain a distribution in the first block of 3 dimensions, explaining 48.9% of the total variance, with a KMO of .848. In the second block we obtain 2 dimensions, explaining 46.3% of the variance, with a KMO of .828. In the third block we obtain 3 dimensions, explaining 55.01% of the variance, with a KMO of .884 (Table 3).

**Table 3:** Barlett’s test of sphericity and KMO for the blocks in the questionnaire.

Block	Sig. In Bartlett’s test of sphericity	KMO. Kaiser-Meyer-Olkin measure of the sample suitability	Number of dimensions	Variance explained
Block I	.000	.848	3	48.9%
Block II	.000	.828	2	46.3%
Block III	.000	.884	3	55.01%

The questionnaire used includes three blocks:

Block I: The first block addressed the perceptions of trainee teachers on how the program had affected their motivation

Block II: The second block of the questionnaire dealt with how satisfied they felt with the program.

Block III: The third block focused on the perception of the learning received in the training program

The results of these tests showed that the questionnaire has an adequate degree of reliability and validity. Descriptive statistical analyses were carried out (minimum, maximum, mean and standard deviation of each of the variables). In addition, mean tests (Student t and single factor ANOVA) were applied for sex and group variables; and nonparametric tests (Mann-Whitney U test) for the sex variable; and Pearson correlations between subscales.

## Results

### Opinion of trainee teachers about the strategies and techniques used in the training program

We can see below (Table 4) the means and standard deviations, as well as the minimum and maximum, of the participants according to the membership group of each of the variables referring to the perception of the strategies used in the study and the grouped variables.

**Table 4:** Descriptive statistics obtained for the variables referring to the evaluation of the strategies used by group.

	Group 1			Group 2			Group 3			Group 4		
	(n = 43)			(n = 53)			(n = 60)			(n = 52)		
Strategy used	M (DT)	Min	Max	M (DT)	Min	Max	M (DT)	Min	Max	M (DT)	Min	Max
Vídeos of the flipped-classroom	4.51 (.77)	3	5	4.23 (.70)	2	5	4.42 (.72)	2	5	4.52 (.65)	3	5
Whole group activities	4.26 (.79)	2	5	4.28 (.72)	2	5	4.50 (.65)	2	5	4.37 (1.01)	1	5
Socrative test	4.72 (.50)	3	5	4.38 (.71)	3	5	4.57 (.59)	3	5	4.54 (.61)	3	5
Score and badges (rewards)	4.49 (.67)	3	5	4.04 (1.24)	1	5	4.23 (.81)	2	5	4.21 (.80)	3	5
Work in small	4.56 (.67)	3	5	4.21 (.99)	1	5	4.45 (.59)	3	5	4.40 (1.03)	1	5
SimulationTeaching Unit	4.73 (.59)	3	5	4.45 (.69)	2	5	4.53 (.62)	2	5	4.46 (.98)	1	5
**Strategy used Total**	4.52 (.46)	2	5	4.26 (.56)	1	5	4.45 (.46)	2	5	4.47 (.54)	1	5

The scores show a very positive evaluation of the strategies used. All items were rated higher than 4 out of 5, and a large part over 4.5. Overall, Group 1 rated the strategies used in the training program most positively while Group 2 gave the lowest rating. At between-group level, when differentiating each of the strategies and techniques it is observed that group 1 values all the items more positively, except that of "Practical activities in the whole group", in which it is the students of Group 3 who award a higher mean score, and "Videos of the flipped-classroom", where Group 4 has a slightly higher mean. At the within-group level, the highest scores are for groups 1 and 2 for the "Simulation" strategies, and for groups 3 and 4 for the "Socrative Test" strategy.

A single factor ANOVA was performed to check for statistically significant differences and the mean differences found between the four groups were not statistically significant.

We present (Table 5) differentiated descriptions of the two strategies/techniques associated with the flipped-classroom (videos and activities in the large group class) and gamification (Socrative test and scores/badges). It can be seen that Group 1 valued the gamification strategies (Socrative test and scores and badges) notably more positively than the rest of the groups. However, Group 1 does not value the techniques associated with flipped-classroom as positively as Groups 3 and 4.

**Table 5:** Descriptive statistics by group membership for the variables referring to the evaluation of the strategies used.

	Group 1			Group 2			Group 3			Group 4		
	(n = 43)			(n = 53)			(n = 60)			(n = 52)		
Strategy used	M (DT)	Min	Max	M (DT)	Min	Max	M (DT)	Min	Max	M (DT)	Min	Max
Flipped-classroom videos/ Whole group practical activites	4.38 (.72)	2	5	4.25 (.60)	2	5	4.45 (.64)	3	5	4.51 (.60)	2	5
Socrative Test/ scores and badges	4.6 (.51)	3	5	4.21 (.36)	2	5	4.40 (.62)	3	5	4.38 (.60)	3	5

A single factor ANOVA was performed to ascertain whether there were statistically significant differences in the assessment of the techniques used associated with the flipped-classroom. The mean differences found between the four groups were not statistically significant. However, a single-factor ANOVA was also performed for the evaluation of the gamification techniques used. The results showed statistically significant differences for Group 1 with respect to Group 2 (Table 6). This group reported a better assessment of the gamification strategies and techniques, with statistically significant differences with respect to Group 2, and with minor differences with the rest of the groups.

**Table 6:** Summary of the ANOVA post hoc tests: Multiple comparisons by groups of the learning perceptions acquired in the training program.

ANOVA					POST HOC (Dunnett’s T3)
	Sum of squares	gl	F	Sig.	
Between groups	15.046	3	2.896	.036	1>2
Within groups	353.334	204			
Total	368.380	207			

As we observed, the descriptive statistics by sex for the perception of strategies variable, with males (26.5%) and females (74.5%) scores very similarly: mean 4.34 (male) versus 4.45 (female).

The Student t test, for independent samples, and the non parametric Mann- Whitney U test were carried out to see if participants’ perceptions of the strategies differed according to sex. In neither case were statistically significant differences found.

### Perception of acquired learning under the training program

We can see (Table 7) the means and standard deviations, as well as the minimum and maximum, of the participants according group membership for each of the variables referring to the perception of learning in the training program and with the grouped items.

**Table 7:** Descriptive statistics for the perceptions of learning acquired during the training program.

	Group 1			Group 2			Group 3			Group 4		
	(n = 44)			(n = 54)			(n = 60)			(n = 52)		
Item	M (SD)	Min	Max	M (SD)	Min	Max	M (SD)	Min	Max	M (SD)	Min	Max
Structure Teachng Unit	4.86 (.35)	4	5	4.59 (.66)	2	5	4.63.(55)	3	5	4.58 (.60)	3	5
Activities and stages	4.91 (.29)	4	5	4.57 (.69)	2	5	4.67 (.51)	3	5	4.63 (.69)	2	5
Evaluation	4.75 (.49)	3	5	4.48 (.72)	2	5	4.52 (.62)	2	5	4.48 (.70)	2	5
Methodology	4.82 (.49)	3	5	4.56 (.60)	3	5	4.72 (.49)	3	5	4.62 (.56)	3	5
**Total Score**	4.83 (.34)	3	5	4.55 (.56)	2	5	4.63 (.42)	2	5	4.57 (.57)	2	5

The scores obtained show a very positive evaluation of the learning acquired in the program. All the items obtained a valuation above 4 out of 5, and all but one exceeded 4.5 (Group 4 rated its learning 4.48). Overall, Group 1 rated its learning most positively and Group 2 the least positively. At between-group level, Group 1 values learning more positively in all variables. At within-group level, the perception is more positive in Groups 1 and 4 in "Activities and phases"; Group 2 students perceive better learning in "Structure of the Didactic Unit" and Group 3 in "Methodology".

In order to analyze whether the perceptions that future teachers have of the learning achieved in the training program based on flipped-classroom and gamification were statistically different, a single-factor ANOVA test was carried out. The results showed statistically significant differences for Group 1 with respect to the rest of the groups: there is a 0.24 difference between group 1 and the rest in learning perception, which is explained because of a higher valuation for methodology and activities by the members of group 1.

In addition, the descriptive statistics for the perceptions of learning acquired during the training program according to participants’ sex show that the mean for females is higher (4.7) than for males (4.43).

In order to analyze whether the differences are significant, both the Student t test for independent samples and the non-parametric Mann-Whitney U test were applied. The results showed statistically significant differences between males and females the overall perception score of their learning, and this was higher in females (Mann-Whitney U = 5.541; p< 0.01; Student t = .000). In the light of the results, female participants perceived that they had learned more.

The learning perception subscale and the techniques/strategies assessment subscale show significant correlations (Correlation = .621; p< 0.000). As we can see (Table 8), the correlations of all items are significant and positive. However, the items of the learning perception subscale show higher correlations among themselves than the items of the techniques/strategies assessment subscale. Correlations between the items of the learning perception subscale range from .515 to .799; while the items of the techniques/strategies assessment subscale range from .174 to .718. With regard to the correlations between the items of both subscales, we observe that they are of magnitudes from weak to moderate, ranging from 251 (Socrative test and Activities) to 453 (UD Simulation and Structure).

**Table 8:** Correlations between the variables of the subscales. Perception of learning and evaluation of techniques/strategies.

		Percepction of learning				Evaluation of techniques/strategies					
		Structure	Activities	Evaluation	Methodology	Flip L videos	Prac Activ	Socrative Test	Score_Rewards	Group work	Simul_UD
Structure	Pearson’s Correlation										
Activities	Pearson’s Correlation	.799**									
	N	210									
Evaluation	Pearson’s Correlation	.629**	.671**								
	N	210	210								
Methodology	Pearson’s Correlation	.515**	.560**	.564**							
	N	210	210	210							
Vídeos_flip class	Pearson’s Correlation	.416**	.364**	.312**	.361**						
	N	206	206	206	206						
Practical activities	Pearson’s Correlation	.308**	.339**	.344**	.292**	.512**					
	N	208	208	208	208	206					
Socrative Test	Pearson’s Correlation	.344**	.251**	.310**	.333**	.509**	.354**				
	N	208	208	208	208	206	208				
Socre_Rewards	Pearson’s Correlation	.366**	.298**	.347**	.288**	.357**	.294**	.482**			
	N	208	208	208	208	206	208	208			
Group Work	Pearson’s Correlation	.329**	.364**	.330**	.314**	.354**	.537**	.230**	.248**		
	N	208	208	208	208	206	208	208	208		
Simul_UD	Pearson’s Correlation	.453**	.438**	.343**	.358**	.407**	.534**	.229**	.174*	.718**	
	N	206	206	206	206	204	206	206	206	206	

**. Correlation is significant at level 0.01 (bilateral)

* correlation is significant at level 0.05 (bilateral).

### Regressions-multivariate analysis with PLS

After analysing the descriptive statistics, the difference between the result variables and the socio-demographic variables, as well as the correlation between subscales, a multivariate regression analysis approach has been used with the Partial least squares regression (PLS regression). The goal is to inspect which variables of the study are more predictive. The partial least squares regression (PLS) is a technique that reduces the predictors to a smaller set of not correlated components and performs a least squares regression on these components, instead of on the original data. Given the fact that the PLS regression models the response variables in a multivariate way, the results could differ significantly from the ones calculated for the response variables individually.

In the Table 9 it can be seen the variables’ importance description for the projection, the standard deviation, as well as the inferior and superior limits for every explanatory variable used in our study.

**Table 9:** Variable Importance in the Projection (VIP).

Variable	VIP	Standard Deviation	Lower Limit (95%)	Upper Limit (95%)
Work in group	1.349	.228	.899	1.799
Gamification strategies	1.249	.207	.841	1.657
Fliiped-classroom strategies	1.225	.142	.946	1.504
Group	.742	.251	.247	1.236
Gender	.627	.316	.003	1.250
Age	.422	.168	.090	.753

**Fig 1:**
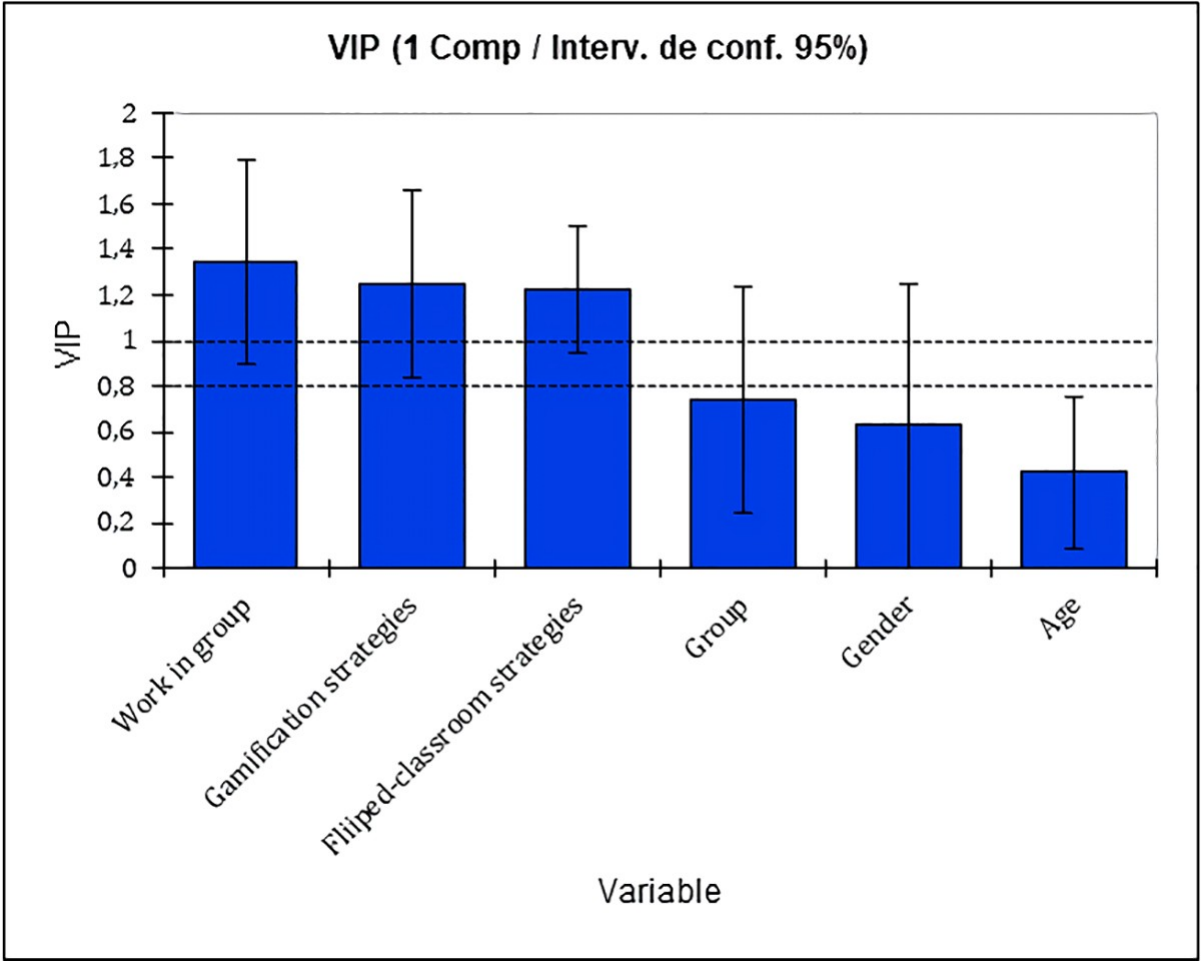
Variable Importance in the Projection (VIP).

As shown in Fig 1, the variables provided by the most optimal values for the projection of our regression model are provided by the teamwork, the gamification strategies and the flipped-classroom strategies, respectively.

**Fig 2:**
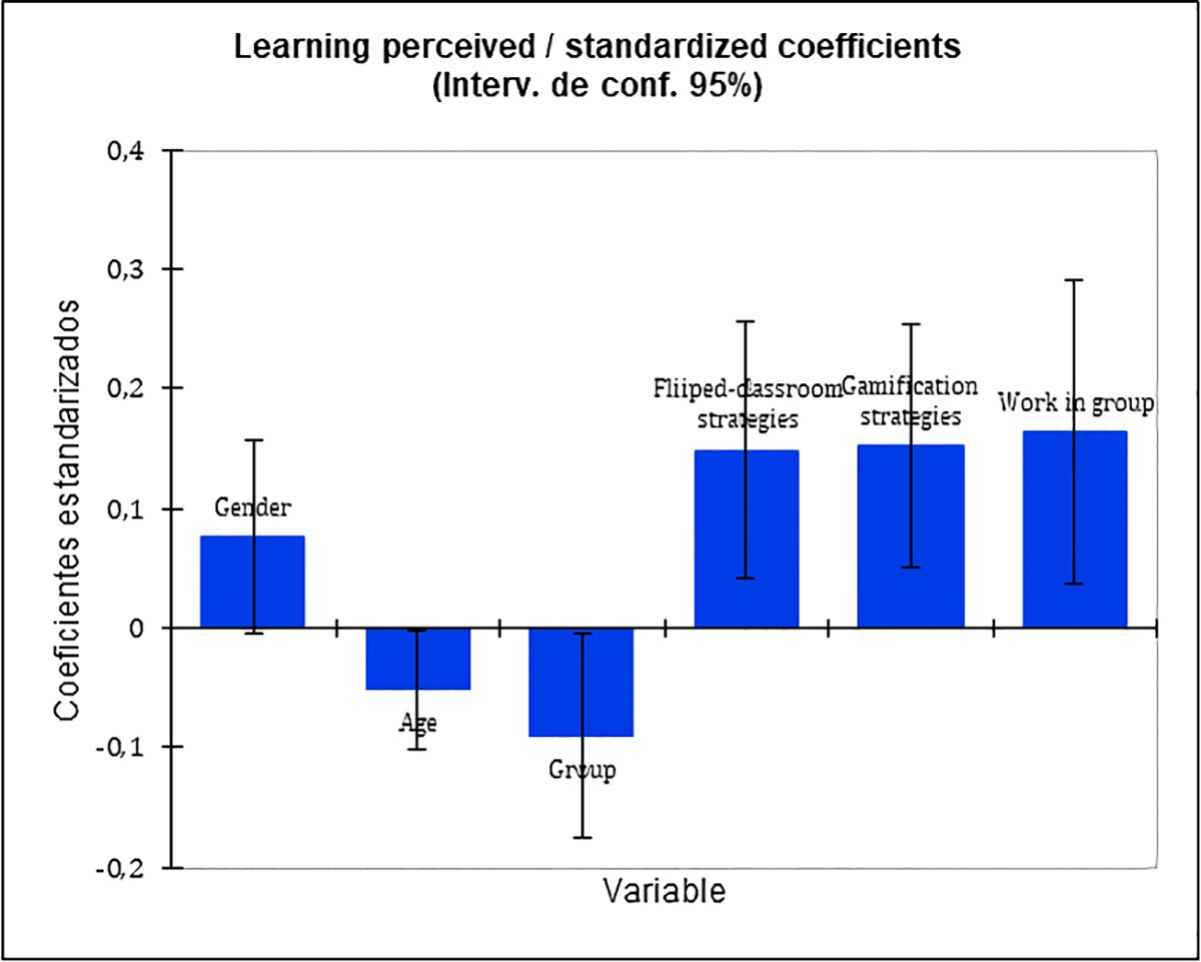
Learning perceived/standardized coefficients.

The importance of these variables in the explanation of the perceived learning can also be noted in Fig 2 on standardized rates. Teamwork is the most influent variable on the perceived learning, followed by gamification strategies, flipped-classroom strategies (with similar values between strategies). With these results we can say that the socio-demographic variables of the study (sex, age and gender) do not have a strong causal relationship with the perceived learning by the students in this training programme. By contrast, in the student’s assessment of the group work, the gamification and flipped-classroom strategies had a notable influence in the perceived learning.

### Results of the future teachers’ learning with regard to the capacity to produce teaching proposals

After knowing the perceptions of the students about their learning, we evaluated the learning outcomes of teachers in training on their ability to propose didactic activities. For it, we used an observation scale that would allow us to assess the structure of these activities, the methodology, the phases and the proposed evaluation. We can see (Table 10) the means and standard deviations, as well as the minimum and maximum, of the participants according to group membership for each of the variables and with the grouped items referring to the learning results for the capacity to propose training activities for the teaching of the social sciences.

**Table 10:** Descriptive statistics for the evaluation of the teaching units.

	Group 1			Group 2			Group 3			Group 4		
	(n = 44)			(n = 14)			(n = 13)			(n = 14)		
Item	M (DT)	Min	Max	M (DT)	Min	Max	M (DT)	Min	Max	M (DT)	Min	Max
Structure of Teaching Unit	4.40 (.70)	3	5	4.57 (.65)	3	5	3–54 (.66)	2	4	4.07 (.83)	2	5
Activities and stages	4.20 (1.23)	1	5	4.93 (.27)	4	5	4.46 (.78)	3	5	4.57 (.51)	4	5
Evaluation	3.90 (.32)	3	4	3.64 (1.22)	2	5	3.69 (.63)	3	5	3.64 (.84)	2	5
Methodology	3.70 (1.16)	1	5	2.79 (2.01)	1	5	4.08 (1.11)	2	5	3.50 (.76)	2	5
**Total score**	4.05 (.74)	1	5	3.98 (.63)	1	5	3.94 (.63)	2	5	3.94 (.57)	2	5

The scores show positive results for the learning acquired in the training program, although with a lower score than the self-perception of t learning. Half of the items received a score over 4 (4.93 out of 5 in the valuation of the activities in Group 2 was the variable with the highest score). The rest of the items exceeded 3.5 out of 5 on average, except for the score received for the methodology in Group 2. Overall, Group 1 had the highest performance in the training program and Group 3 the lowest, although the difference was not very great. At between-group level, the averages between all the groups showed few differences. Nevertheless, the best results were obtained in "Structure of the Teaching Unit" in Group 1; in "Activities and phases" in Group 2 and in "Evaluation" and in "Methodology" in Group 3. At the within-group level, the highest scores were from Group 1 in "Structure of the Teaching Unit", and Groups 2, 3 and 4 in "Activities and stages".

A single factor ANOVA was run to test for significant differences and the differences in means detected were not significant.

Table 11 collects the descriptive statistical data from the learning perception score and the performance of the Didactic Unit. The sample difference between the learning perception and performance questionnaires obtained in the Didactic Unit evaluation is due to the fact that in the latter the evaluation was conducted in a group.

**Table 11:** Descriptive statistics for future teachers’ perceptions about learning and acquired outcomes.

	Questionnaire perception mean	Evaluation teaching units mean	Difference	Student's t-test	Significance (Two-tailed test)
	N = 210	N = 51			
Item	M (SD)	M (SD)			
Learning about a Didactic Unit Structure^2^	4.30 (.862)	4.14 (.800)	,160	1,260	.211
Learning about the phases of a Didactic Units’ activities^1^	4.37 (,886)	4,57 (.755)	-,200	-1,485	.139
Learning about the evaluation tools of a Didactic Unit^2^	4.60 (.673)	3.71 (.832)	.890	7.092	< .001
Learning about the different active teaching methods in social sciences^2^	4.69 (.584)	3.49 (1,405)	.201	5.975	< .001

^1^Equal variance is assumed

^2^Equal variance is not assumed

On one side, if we compare the scores on Table 11 on a descriptive level, it can be observed that the higher scores are obtained on the perception of the achieved learning (regarding the perception of the Didactic Unit, the evaluation methods of the Didactic Unit and the active teaching methods in social sciences’ structures). However, regarding the phases of a Didactic Units’ activities, the performance levels are higher than what is perceived.

To analyse if the observed differences at a descriptive level are statistically significant, a t of Student was used for independent samples, observing that there are statistically significant differences in the learning perception regarding the achieved learning (in terms of both evaluation tools of a Didactic Unit and the learning of different active teaching methods in social sciences).

## Discussion and conclusions

From the data, the students had a very positive opinion of the strategies and techniques used in the training program. Increased participation, greater autonomy and the ability to tackle different learning styles [36–38, 59, 75]; as well better commitment towards the learning [20, 57, 76, 77] are some of the factors that would explain this. Although there were no statistically significant differences in terms of groups and gender overall, Group 1 did score gamification strategies higher.

As regards perception of learning, the results are again very positive. This perception of learning expressed by the students themselves is in line with other research on the use of gamification and the flipped-classroom [49, 78–80]. In this case there were significant differences. Group 1 students and female participants perceived that they had learned more. There is a wealth of literature that has addressed differences in the perception of the use of technology and digital literacy according to gender [81, 82]. In this training program, in which ICTs played an important role, it was women who showed a better perception of learning and a greater appreciation of the program. These results differ from a large part of the studies, which indicate notable differences in the use and usefulness of ICT [83]. We interpret the women's more positive opinions of the program and the techniques as being related more to their innovative potential than to the use of ICT. The supposed gender digital divide [84] must, therefore, be taken with extreme caution, and the results should be analyzed from different conceptions of what innovation means [85]. The differences for Group 1 have an explanation first in their special characteristics (bilingual group), and second in their greater valuation of techniques and strategies linked to gamification. These techniques are related to the increase in student motivation [9]. The data seem to indicate that this motivation generally supposed a greater self-perception of learning. This was a group with a higher academic level than the rest of the groups, and valued the motivation techniques more positively.

The analysis of the learning outcomes through the evaluation of the teaching units designed by the students shows satisfactory results. However, this score was lower than the self-perception of learning. The students believed that they had learned more than the final results showed after the teachers' evaluations. Group 1 had the best results, although with a statistically insignificant difference with the rest of the groups. The structure of the training units and the activities were the items with the best outcome among the teachers in training. On the other hand, the methodological justification at the theoretical level did not have such satisfactory results.

From training in university contexts, one must begin to understand that international studies and reports are proposing that these key trends be adopted in the short term, leading to a change in practices in educational contexts. In our study, students valued very positively the use of previous videos through the flipped-classroom method, as in other studies [46]. This allowed to change the traditional lesson and to introduce activities of work in group. The use of in-class time to do these activities resulted in more positive feedback from students. Student learning, as determined by Teaching units designed, was affected positively [45]. The good evaluation of the program was also increased thanks to the use of gamification techniques. If the use of flipped-classroom was a methodological change in which students acquired a more active role, the use of gamification kept motivation high with daily work [20]. University education and initial teacher training must adapt to these challenges and the demands of today's society, taking into account the emerging trends that professionals will encounter in their immediate future [86, 87].

Not all published experiences on gamification reflect these positive results [19, 38]. Hence, from the conclusions of these experiences, we interpret that the satisfactory data obtained in our research are due to our students’ being informed of the working method from the outset [37] and it was accepted once the educational objectives associated with the program had been established. The learners involved themselves in the learning process by watching the videos and so arrived prepared in the classroom. In tandem with the dynamics of the flipped-classroom and gamification, we could also count on the cooperative work to fix learning and internal motivation [9].

According to the data obtained, the teachers in training acquired specific competences in the proposal of training activities for teaching social sciences. However, learning on the theoretical conceptualization capacity of these proposals was more superficial learning. This undoubtedly seems to be one of the weak points of the program, with more emphasis placed on technical and design skills. The program needs to be reviewed in order to provide future teachers with a greater theoretical framework on which to base their training proposals. However, in order to corroborate the effects of this program, more in-depth evaluative research is needed with larger numbers of learners and in different contexts.

## Supporting information

S1 Checklist (DOCX)

S1 Data (DOCX)

S1 File Evaluation scale for Teaching Units (TU). (DOCX)

## References

[1] González G, Skultety L. Teacher learning in a combined professional development intervention, TEACH TEACH EDUC. 2018; 71: 341–354. 10.1016/j.tate.2018.02.003

[2] Barnes N, Fives H, Dacey C. U.S. teachers' conceptions of the purposes of assessment. TEACH TEACH EDUC. 2017;65:107–116. 10.1016/j.tate.2017.02.017

[3] Gómez CJ, Rodríguez RA, Mirete AB. Percepción de la enseñanza de la historia y concepciones epistemológicas. Una investigación con futuros maestros. RCED. 2018;29 (1):237–250. Spanish. 10.5209/RCED.52233

[4] Korthagen AJ. Situated Learning Theory and the Pedagogy of Teacher Education: Towards an Integrative View of Teacher Behavior and Teaching Learning. TEACH TEACH EDUC. 2010;26(1):98–106. 10.1016/j.tate.2009.05.001

[5] König J, Pflanz B. Teacher knowledge associated with performance? On the relationship between teachers’ general pedagogical knowledge and instructional quality. EJTE.39(4): 419–436. 10.1080/02619768.2016.1214128

[6] Oliveira C, Lopes J, Spear-Swerling L. Teachers’ academic training for literacy instruction. EJTE. 2019;42(3):315–334. 10.1080/02619768.2019.1576627

[7] Hakak S, Noor FNM, Ayub M, Hannyzurra A, Hussin, N. Ahmed E, et al Cloud-assisted gamification for education and learning–Recent advances and challenge. CEE. 2019;74:22–34. 10.1016/j.compeleceng.2019.01.002

[8] Vahedi Z, Zannella L, Want SC. Students’ use of information and communication technologies in the classroom: Uses, restriction, and integration. ALHE. 2019 10.1177/1469787419861926

[9] Da Rocha L, Sandro A, De Melho I. Effectiveness of gamification in the engagement of students. Comput Human Behav. 2016; 58: 48–63. 10.1016/j.chb.2015.11.021

[10] Ding L. Applying gamifications to asynchronous online discussions: A mixed methods study. Comput Human Behav. 2019;91:1–11. 10.1016/j.chb.2018.09.022

[11] Fitz-Walter Z, Tjondronegoro D, Wyeth P. A gamified mobile application for engaging new students at university orientation. Paper presented at: OzCHI 2012. Proceedings of the 24th Australian Computer-Human Interaction Conference; 2012 Nov 26–30; Melbourne, Australia. New York: ACM; 2012. p. 138–141. https://doi.acm.org/10.1145/2414536.2414560

[12] Attali Y, Arieli-Attali M. Gamification in assessment: do points affect test performance? Comput Educ. 2015;83:57–63. 10.1016/j.compedu.2014.12.012

[13] Werbach K, Hunter D. For the win: How game thinking can revolutionize your business. Philadelphia: Wharton Digital Press; 2012.

[14] McGonigal J. Reality is broken: Why games make us better and how they can change the world. New York: Penguin; 2011.

[15] Liu Y, Alexandrova T, Nakajima T. Gamifying intelligent environments. Paper presented at: MM'11 Proceedings of the 2011 international ACM workshop on ubiquitous meta user interfaces; 2011 Dec 1; Scottsdale, USA. New York: ACM Computer Society; 2011 p. 7–12. 10.1145/2072652.2072655

[16] Hamari J, Koivisto J, Sarsa H. Does Gamification Work?–A Literature Review of Empirical Studies on Gamification. Paper presented at: HICSS.2014 Proceedings of the 47th Hawaii International Conference on System Sciences; 2014 Jan 6–9; Hawaii, USA. New York: IEEE Computer Society; 2014 p. 3025–3034. 10.1109/HICSS.2014.377

[17] Whitton N, Langan M. Fun and games in higher education: an analysis of UK student perspectives. TiHE. 2018;23 10.1080/13562517.2018.1541885

[18] Ortega Sánchez D, Gómez Trigueros IM (2019) Gamification, social problems, and gender in the teaching of social sciences: Representations and discourse of trainee teachers. PLoS ONE; 14(6): e0218869 10.1371/journal.pone.0218869 31242248 PMC6594637

[19] Hanus M, Fox J. Assessing the effects of gamification in the classroom: A longitudinal study on intrinsic motivation, social comparison, satisfaction, effort, and academic performance. Comput Educ. 2015;80:152–161. 10.1016/j.compedu.2014.08.019

[20] Mekler ED, Brühlmann F, Tuch AN, Opwis K. Towards understanding the effects of individual gamification elements on intrinsic motivation and performance. Comput Human Behav. 2017;71:525–534. 10.1016/j.chb.2015.08.048

[21] Sailer M, Hense JU, Mayr SK, Mandl H. How gamification motivates: an experimental study of the effects of specific game design elements on psychological need satisfaction. Comput Human Behav. 2017;69:371–380. 10.1016/j.chb.2016.12.033

[22] Chapman JR, Rich PJ. Does educational gamification improve students’ motivation? If so, which game elements work best? JEFB. 2018; 93(7): 315–322. 10.1080/08832323.2018.1490687

[23] Urh M, Vukovic G, Jereb E, Pintar R. The model for introduction of gamification into e-learning in higher education. Procedia Soc Behav Sci. 2015;197:388–397. 10.1016/j.sbspro.2015.07.154

[24] Spence I, Feng J. Video Games and Spatial Cognition. Rev. Gen. Psychol. 2010;2(10):92–104. 10.1037/a0019491

[25] Landers NR, Callan RC. Casual Social Games as Serious Games: The Psychology of Gamification in Undergraduate Education and Employee Training In Ma M, Oikonomou A, Jain L, editors. Serious Games and Edutainment Applications. London: Springer-Verlag, 2011 pp. 399–423. 10.1007/978-1-4471-2161-9_20

[26] Kim BD, Shi M, Srinivasan K. Reward programs and tacit collusion. Mark. Sci. 2001;20:99–120. 10.1287/mksc.20.2.99.10191

[27] Silva R, Rodrigues R, Leal C, Leal CT. Gamification in Accounting Higher Education: Stepwise and OLS regressions. Paper presented at: TAKE 2017. Proceedings of the 2017 International Conference Theory and Applications in the Knowledge Economy; 2017 Jul 12–14; Zagreb, Croatia: Electronic format; 2017 p. 444–455. Retrieved from https://www.researchgate.net/profile/Judy_Smetana/publication/322686268_Organizational_Culture_and_Leadership's_Impact_on_a_Safety_Program_Change_Model/links/5a68c737aca2728d0f5e04dd/Organizational-Culture-and-Leaderships-Impact-on-a-Safety-Program-Change-Model.pdf

[28] Sykes G, Bird T, Kennedy M. Teacher education: Its problems and some prospects. J. Teach. Educ. 2010;61(5):464–476. 10.1177/0022487110375804

[29] Hao Y, Lee K. Teaching in flipped classrooms: Exploring pre-service teachers' concerns. Comput Human Behav. 2016; 57: 250–260. 10.1016/j.chb.2015.12.022

[30] Vaughan M. Flipping the learning: an investigation into the use of the flipped classroom model in an introductory teaching course. ERP. 2014;41(1):25–41. Retrieved July 26, 2019 from https://www.learntechlib.org/p/153305/

[31] Abeysekera L, Dawson P. Motivation and cognitive load in the flipped classroom: definition, rationale and a call for research. HERD. 2015; 34(1): 1–14. 10.1080/07294360.2014.934336

[32] Koehler M, Mishra P. What is technological pedagogical content knowledge (TPACK)? CITE. 2009;1(9):60–70. Retrieved from https://www.citejournal.org/volume-9/issue-1-09/general/what-is-technological-pedagogicalcontent-knowledge

[33] Pamuk S. Understanding preservice teachers’ technology use through TPACK framework. JCAL. 2011;28(5):425–439. 10.1111/j.1365-2729.2011.00447.x

[34] Hofer M, Grandgenett N. TPACK Development in teacher education. A longitudinal study of preservice teachers in a Secondary M.A.Ed. Program. JRTE. 2012;45(1):83–106. 10.1080/15391523.2012.10782598

[35] O’Flaherty J, Phillips C. The use of Flipped Classrooms in Higher Education: A Scoping Review. INTERNET HIGH EDUC. 2015; 25: 85–95. 10.1016/j.iheduc.2015.02.002

[36] Steen-Utheim AT, Foldnes N. A qualitative investigation of student engagement in a flipped classroom. TiHE. 2018; 23(3): 307–324. 10.1080/13562517.2017.1379481

[37] Gilboy MB, Heinerichs S, Pazzaglia G. Enhancing student engagement using the flipped classroom. J Nutr Educ Behav. 2015; 47(1): 109–114. 10.1016/j.jneb.2014.08.008 25262529

[38] Burke A, Fedorek B. Does flipping promote engagement?: A comparison of a traditional, online and flipped class. ALHE. 2017; 18(1): 11–24. 10.1177/1469787417693487

[39] Cochran-Smith M, Zeichner KM. Studying teacher education: The report of the AERA panel on research and teacher education. New York: Routledge; 2005.

[40] Darling-Hammond L, Bransford JD. Preparing teachers for a changing world: What teachers should learn and be able to do. San Francisco, CA: Jossey-Bass; 2005.

[41] König J, Ligtvoet R, Klemenz S, Rothland M. Effects of opportunities to learn in teacher preparation on future teachers’ general pedagogical knowledge: Analyzing program characteristics and outcomes. Stud Educ Eval. 2017;53:122–133. 10.1016/j.stueduc.2017.03.001

[42] Floden R. Learning what research says about teacher preparation In Feuer MJ, Berman AI, Atkinson RC, editors. Past as prologue: The national academy of education at 50 Members reflect. Washington, DC: National Academy of Education; 2015 p. 279–284.

[43] Blömeke S, Suhl U, Kaiser G, Döhrmann M. Family background, entry selectivity and opportunities to learn: What matters in primary teacher education? An international comparison of fifteen countries. TEACH TEACH EDUC. 2012;28(1):44–55. 10.1016/j.tate.2011.08.006

[44] Chen HYL, Chen NS. Design and evaluation of a flipped course adopting the holistic flipped classroom approach. Paper presented at: IEEE.2014 Proceedings of the 14th International Conference on Advanced Learning Technologies; 2014 7 7–10; Athens, Greece: Institute of Electrical and Electronics Engineers (IEEE); 2014.p. 627–631. 10.1109/ICALT.2014.183

[45] Velegol SB, Zappe SE, Mahoney E. The Evolution of a Flipped Classroom: Evidence-Based Recommendations. AEE. 2015;4(3):1–37.

[46] Young TP, Bailey CJ, Guptill M, Thorp AW, Thomas TL. The flipped classroom: a modality for mixed asynchronous and synchronous learning in a residency program. West J Emerg Med. 2014;15(7):938–944. 10.5811/westjem.2014.10.23515 25493157 PMC4251258

[47] Findlay-Thompson S, Mombourquette P. Evaluation of a flipped classroom in an undergraduate business course. BEA. 2014;6(1):63–71.

[48] Seaborn K, Fels DI. Gamification in theory and action: A survey. Int J Hum Comput Stud. 2015; 74:14–31. 10.1016/j.ijhcs.2014.09.006

[49] Zou D. Gamified flipped EFL classroom for primary education: student and teacher perceptions. J. Comput. Educ.2020 10.1007/s40692-020-00153-w

[50] Dicheva D, Dichev C, Gennady A, Angelova G. Gamification in Education: a systematic mapping study. EDUC TECHNOL SOC. 2015;18(3):75–88.

[51] Kamasheva AV, Valeev ER, Yagudin RK, Maksimova KR. Usage of Gamification theory for increase motivation of employees. Mediterr J Soc Sci. 2015;6(15):77–80. 10.5901/mjss.2015.v6n1s3p77

[52] Oliván-Blázquez B, Masluk B, Gascon S, Fueyo-Díaz R, Aguilar-Latorre A, Artola-Magallón I, et al The use of flipped classroom as an active learning approach improves academic performance in social work: A randomized trial in a university. 2019;PLoS ONE;14(4): e0214623 10.1371/journal.pone.0214623 30947270 PMC6448877

[53] Shih W, Tsai C. Effect of Flipped Classroom with BOPPPS Model on Learners' Learning Outcomes and Perceptions in a Business Etiquette Course. Asia-Pacific Edu Res. 2019 10.1007/s40299-019-00475-z

[54] Topper A, Lancaster S. Online graduate educational technology program: An illuminative evaluation. Stud Educ Eval. 2016; 51: 108–115. 10.1016/j.stueduc.2016.10.002

[55] Morganti L, Pallavicini F, Cadel E, Candeleri A, Archetti F, Mantovani F. GaminG for Earth: serious games and gamification to engage consumers in pro-environmental behaviours for energy efficiency. ERSS.2017;29:95–102. 10.1016/j.erss.2017.05.001

[56] Cózar-Gutiérrez R, Sáez-López JM. Game-based learning and gamification in initial teacher training in the social sciences: an experiment with MinecraftEdu. ETHE.2016;13 10.1186/s41239-016-0003-4

[57] Zainuddin Z. Student’s learning performance and perceived motivation in gamified flipped-class instruction. Comput Educ. 2018;126:75–88. 10.1016/j.compedu.2018.07.003

[58] Zhamanov A, Sakhiyeva Z. Implementing flipped classroom and gamification teaching methods into computers networks subject, by using cisco networking academy. Paper presented at: ICECCO.2015 Proceedings of the Twelve International Conference on Electronics Computer and Computation; 2015 9 27–30; Almaty, Kazakhstan: Institute of Electrical and Electronics Engineers (IEEE); 2015.p. 1–4. 10.1109/ICECCO.2015.7416890

[59] Hasan A, Sezer K, Fezile O. Effects of the gamification supported flipped-classroom model on the attitudes and opinions regarding game-coding education. iJET.2018;13(1):109–123. 10.3991/ijet.v13i01.7634

[60] Huang B, Hew KF, Lo CK. Investigating the effects of gamification-enhanced flipped learning on undergraduate students’ behavioral and cognitive engagement. Interact. Learn. Environ. 2019;27:1106–1126. 10.1080/10494820.2018.1495653

[61] Adler SA. The education of Social Studies Teachers In Levstik L.S, Tyson CA, editors. Handbook of Research in Social Studies Education. New York: Routledge; 2008 p 329–351.

[62] Wittrock MC. La investigación en la enseñanza III Profesores y alumnos. Barcelona: Paidós Educador/MEC; 1990.

[63] Yilmaz K. Social Studies Teachers' Conceptions of History: Calling on Historiography. J Educ Res. 2008;101(3):158–176. 10.3200/JOER.101.3.158-176

[64] Evans RW. Lessons from history: Teacher and student conceptions of the meaning of history. Theory Res Soc Educ. 1988;16(3):203–225. 10.1080/00933104.1988.10505565

[65] Teixes-Argilés F. Gamificación: fundamentos y aplicaciones. Barcelona: Editorial UOC S.L; 2015.

[66] Çeker E, Özdamh F. What “gamification” is and what it’s not. Eur. J. Contemp. Educ. 2017;6(2):221–228. 10.13187/ejced.2017.2.221

[67] Han-Huey C, Kofinas A, Luo J. Enhancing student learning experience with technology-mediated gamification: An empirical study. Comput Educ. 2018;121:1–17. 10.1016/j.compedu.2018.01.009

[68] De Marcos L, García-López E, García-Cabot A. On the effectiveness of game-like and social approaches in learning: Comparing educational gaming, gamification and social networking. Comput Educ. 2016; 95: 99–113. 10.1016/j.compedu.2015.12.008

[69] Gestsdóttir SM, Van Boxtel C, and Van Drie J. Teaching historical thinking and reasoning: Construction of an observation instrument. BERJ. 2018;44(6):960–981. 10.1002/berj.3471

[70] Gómez CJ, Miralles P. ¿Pensar históricamente o memorizar el pasado? La evaluación de los contenidos históricos en la educación obligatoria en España. rev. estud. soc. 2015;52:52–68. Spanish. 10.7440/res52.2015.04

[71] Van Straaten D, Wilschut A, Oostdam R. Measuring students’ appraisals of the relevance of history: The construction and validation of the Relevance of History Measurement Scale (RHMS). Studies in Educational Evaluation. 2018;56:102–111. 10.1016/j.stueduc.2017.12.002

[72] González J, Pazmiño M. Cálculo e interpretación del Alfa de Cronbach para el caso de validación de la consistencia interna de un cuestionario, con dos posibles escalas tipo Likert. Revista Publicando. 2015;2(1):62–77. Spanish. Retrieved from https://revistapublicando.org/revista/index.php/crv/article/view/22

[73] Oviedo HC, Campo‐Arias A. Aproximación al uso del coeficiente alfa de Cronbach; An Approach to the Use of Cronbach's Alfa. *Rev*. *colomb*. *Psiquiatr*. 2005;34(4):572‐580. Spanish. Retrieved July 26, 2019, from http://www.scielo.org.co/scielo.php?script=sci_arttextandpid=S0034-74502005000400009andlng=enandtlng=es

[74] Lloret-Segura S, Ferreres-Traver A, Hernández-Baeza A, Tomás-Marco I. Exploratory Item Factor Analysis: A practical guide revised and up-dated. Annals of Psychology. 2014;30(3):1151–1169. 10.6018/analesps.30.3.199361

[75] González D, Jeong JS, Rodríguez DA. Performance and perception in the flipped learning model: an initial approach to evaluate the effectiveness of a new teaching methodology in a general science classroom. JSET. 2016;25(3):450–459. 10.1007/s10956-016-9605-9

[76] Hamari J. Do badges increase user activity? A field experiment on the effects of gamification. Comput Human Behav. 2017;71:469–478. 10.1016/j.chb.2015.03.036

[77] Yildirim I. The effects of gamification-based teaching practices on student achievement and students' attitudes toward lessons. The Internet and Higher Education. 2017;33:86–92. 10.1016/j.iheduc.2017.02.002

[78] Borrás O, Martínez M, Fidalgo Á. New challenges for the motivation and learning in engineering education using gamification in MOOC. ijee. 2016;32(1):501–512.

[79] Love B, Hodge A, Grandgenett N, Swift, A. Student learning and perceptions in a flipped linear algebra course. IJMEST. 2014; 45(3), 317–324. 10.1080/0020739X.2013.822582

[80] Samuel ML. Flipped pedagogy and student evaluations of teaching. ALHE. 2019 10.1177/1469787419855188

[81] Boeve-de Pauw J, Jacobs, K Van Petegem P. Gender Differences in Environmental Values: An Issue of Measurement? Environ Behav. 2014;46(3):373–397. 10.1177/0013916512460761

[82] Inglis CJ, Marzo JC, Castejon JL, Nuñez JC, Valle A, Garcia-Fernandez JM, et al Factorial invariance and latent mean differences of scores on the achievement goal tendencies questionnaire across gender and age in a sample of Spanish students. Learn Individ Differ. 2011;21(1):138–143. 10.1016/j.lindif.2010.10.008

[83] Park C, King D, Cho S, Han HJ. Adoption of multimedia technology for learning and gender difference. Comput Human Behav. 2019; 92: 288–296. 10.1016/j.chb.2018.11.029

[84] Wilfred WF, Lau A, Yuen HK. Factorial invariance across gender of a perceived ICT literacy scale. Learn Individ Differ. 2015; 41: 79–85. 10.1016/j.lindif.2015.06.001

[85] Edwards-Schachters M. The nature and variety of innovation. Int. J. Innov. Stud. 2018;2:65–79. 10.1016/j.ijis.2018.08.004

[86] Miralles P, Gómez CJ, Monteagudo J. Perceptions on the use of ICT resources and mass-media for the teaching of History. A comparative study among future teachers of Spain-England. Educ XX1. 2019;22(2):187–211. 10.5944/educxx1.21377

[87] Miralles P, Gómez CJ, Arias VB, Fontal O. Digital resources and didactic methodology in the initial training of history teachers. Comunicar. 2019;61:45–56. 10.3916/C61-2019-04

