# Correction: Evaluation of a gamification and flipped-classroom program used in teacher training: Perception of learning and outcome

**DOI:** 10.1371/journal.pone.0241892

**Published:** 2020-10-29

**Authors:** Cosme J. Gómez-Carrasco, José Monteagudo-Fernández, Juan R. Moreno-Vera, Marta Sainz-Gómez

An additional affiliation is missing for the third author. Juan R. Moreno-Vera is also affiliated with Universidad Autónoma de Chile, Providencia, Chile.

The ORCID iDs are missing for the first, third, and fourth authors. Please see the authors’ respective ORCID iDs here:

- Author Cosme J. Gómez-Carrasco’s ORCID iD is: 0000-0002-9272-5177 (https://orcid.org/0000-0002-9272-5177).
- Author Juan R. Moreno-Vera’s ORCID iD is: 0000-0002-5395-5981 (https://orcid.org/0000-0002-5395-5981).
- Author Marta Sainz-Gómez’s ORCID iD is 0000-0002-5573-9748 (https://orcid.org/0000-0002-5573-9748).
