# Supplementary material for: Evaluation of a gamification and flipped-classroom program used in teacher training: Perception of learning and outcome
Source: PLoS One. 2020 Jul 16;15(7):e0236083. doi: 10.1371/journal.pone.0236083 (PMC7365436; doi:10.1371/journal.pone.0236083)
Supplement: S1 Checklist — (DOCX) [file pone.0236083.s001.docx]

IMPLEMENTATION CHECKLIST

ACTIVITIES

1. Flipped-Classroom related activities

| Activity | Done |
| --- | --- |
| To upload the video elaborated by the teacher with theoretical contents, a week before we start to work with these specific contents |  |
| To upload complementary documents one week in advance |  |
| To upload to the Virtual Classroom the indications needed to discuss the complementary documents and video |  |
| To start the lesson discussing the contents of the video and the complementary documents |  |
| After the discussion, to make group activities about the video content (case studies, simulations, font analysis, etc.) |  |
| To finish the lesson with a discussion about the group activities done |  |

1. Gamification activities

| Activity | Done |
| --- | --- |
| Creation of small groups (3-6 people per group) for gamified work |  |
| To explain the students the badges and weekly awards for the three teams with better average |  |
| To make a Socrative test at the beginning of each lesson to check the content learning through the video |  |
| To make a Socrative test at the end of each lesson to check the content learning through the group activities |  |
| To publish in the Virtual Classroom the weekly ranking for badges acquisition |  |
| To publish in the Virtual Classroom the final ranking for awards acquisition |  |
