# Supplementary material for: Evaluation of a gamification and flipped-classroom program used in teacher training: Perception of learning and outcome
Source: PLoS One. 2020 Jul 16;15(7):e0236083. doi: 10.1371/journal.pone.0236083 (PMC7365436; doi:10.1371/journal.pone.0236083)
Supplement: S1 Data — (DOCX) [file pone.0236083.s002.docx]

***IDENTIFICATION DATA***

| **Sex**: | Woman | ❒ |  | Man | ❒ |  |
| --- | --- | --- | --- | --- | --- | --- |
|  |  |  |  |  |  |  |
| **Age**: ………….  **Group** ……. | | | | | | |

| Coming up next, we show some affirmations. We asked you to fill this questionaire. Check in the box the better answer that fits on you, taking into account that 1= strongly disagree, 2 = disagree; 3 = neither agree nor disagree; 4 = agree; 5 = strongly agree.  **BLOCK 1: Opinion about the influence of the program “Gamification & Flipped Classroom” on your motivation** | Strongly disagree | Disagree | Neithe agree nor disagree | Agree | Strongly agree |  |
| --- | --- | --- | --- | --- | --- | --- |
| 1. Program motivates me to discover new active methodologies to teach Social Sciences | 1 | 2 | 3 | 4 | 5 |  |
| 2. Program improved my motivation on classroom efforts | 1 | 2 | 3 | 4 | 5 |  |
| 3. My motivation improved because the program conected with my future teaching experience | 1 | 2 | 3 | 4 | 5 |  |
| 4. Program motivates me to reach better qualifications | 1 | 2 | 3 | 4 | 5 |  |
| 5. Rankings & qualifications proposed on gamification improved my motivation | 1 | 2 | 3 | 4 | 5 |  |
| 6. Ranking awards (badges) improved my motivation | 1 | 2 | 3 | 4 | 5 |  |
| 7. My motivation on the program was, essentially, passing the subject | 1 | 2 | 3 | 4 | 5 |  |

**8. Valuate from 1 (less relevant) to 5 (more relevant) the role of technics/resources/strategies on your motivation**

| Flipped-Classroom videos | 1 | 2 | 3 | 4 | 5 |
| --- | --- | --- | --- | --- | --- |
| Practical activities in full group | 1 | 2 | 3 | 4 | 5 |
| Socrative tests | 1 | 2 | 3 | 4 | 5 |
| Group qualifications & badges (awards) | 1 | 2 | 3 | 4 | 5 |
| Small group work to elaborate didactic units | 1 | 2 | 3 | 4 | 5 |
| Didactic unit simulation | 1 | 2 | 3 | 4 | 5 |

| **BLOCK 2: Opinion about the influence of the program “Gamification & Flipped Classroom” on your satisfaction** | Strongly disagree | Disagree | Neither agree nor disagree | Agree | Strongly agree |  |
| --- | --- | --- | --- | --- | --- | --- |
| 9. I am satisfied with my role as student in the program | 1 | 2 | 3 | 4 | 5 |  |
| 10. I am satisfied with the role of teachers in the program | 1 | 2 | 3 | 4 | 5 |  |
| 11. I am satisfied with group management done by the teacher | 1 | 2 | 3 | 4 | 5 |  |
| 12. I am satisfied with the work of my colleagues to design the didactic unit in small groups | 1 | 2 | 3 | 4 | 5 |  |

**13. Valuate from 1 (less relevant) to 5 (more relevant) your satisfaction with the role of technics/resources/strategies on the “Gamification & Flipped Classroom program”**

| Flipped-Classroom videos | 1 | 2 | 3 | 4 | 5 |
| --- | --- | --- | --- | --- | --- |
| Practical activities in full group | 1 | 2 | 3 | 4 | 5 |
| Socrative tests | 1 | 2 | 3 | 4 | 5 |
| Group qualifications & badges (awards) | 1 | 2 | 3 | 4 | 5 |
| Small group work to elaborate Didactic Units | 1 | 2 | 3 | 4 | 5 |
| Didactic Unit simulation | 1 | 2 | 3 | 4 | 5 |

| **BLOCK 3: Opinion about the influence of the program “Gamification & Flipped Classroom” on your learning effectiveness** | Strongly disagree | Disagree | Neither agree nor disagree | Agree | Strongly agree |  |
| --- | --- | --- | --- | --- | --- | --- |
| 14. I learned the structure and elements of a didactic unit | 1 | 2 | 3 | 4 | 5 |  |
| 15. I learned the stages of a didactic unit | 1 | 2 | 3 | 4 | 5 |  |
| 16. I learned the processes, technics and evaluation instrument of a didactic unit | 1 | 2 | 3 | 4 | 5 |  |
| 17. I learned why a methodological change in Social Sciences education is needed | 1 | 2 | 3 | 4 | 5 |  |
| 18. I learned different active methodologies in Social Sciences teaching | 1 | 2 | 3 | 4 | 5 |  |
| 19. I learned different ways of using media in a classroom | 1 | 2 | 3 | 4 | 5 |  |
| 20. I learned different ways to use primary fonts and heritage | 1 | 2 | 3 | 4 | 5 |  |
| 21. I learned different ways to use ITC in Social Sciences education | 1 | 2 | 3 | 4 | 5 |  |

**22. Valuate from 1 (less relevant) to 5 (more relevant) the role of technics/resources/strategies on the “Gamification & Flipped Classroom program” for your learning effectiveness**

| Flipped-Classroom videos | 1 | 2 | 3 | 4 | 5 |
| --- | --- | --- | --- | --- | --- |
| Practical activities in full group | 1 | 2 | 3 | 4 | 5 |
| Socrative tests | 1 | 2 | 3 | 4 | 5 |
| Group qualifications & badges (awards) | 1 | 2 | 3 | 4 | 5 |
| Small group work to elaborate didactic units | 1 | 2 | 3 | 4 | 5 |
| Didactic unit simulation | 1 | 2 | 3 | 4 | 5 |
