# Supplementary material for: Evaluation of a gamification and flipped-classroom program used in teacher training: Perception of learning and outcome
Source: PLoS One. 2020 Jul 16;15(7):e0236083. doi: 10.1371/journal.pone.0236083 (PMC7365436; doi:10.1371/journal.pone.0236083)
Supplement: S1 File — (DOCX) [file pone.0236083.s003.docx]

Evaluation scale for Teaching Units (TU)

| **Criteria**  **Items** | **1-2** | **3** | **4-5** | **Score** |
| --- | --- | --- | --- | --- |
| **Suitability of the structure of the Teaching Unit** | TU doesn’t contain all the elements or they are confused | TU contains all the elements but they are not linked | TU contains all the elements and they are adequate | 0-1 |
| **Methodological suitability** | TU doesn’t use the theoretical frame of the subject or just uses personal opinions without theoretical discussion | TU uses the theoretical frame of the subject but it is confused and not linked. There are content errors or TU doesn’t use the required papers of the subject | Correct use of the theoretical frame of the subject, using the required papers proposed by teachers | 0-2 |
| **Relevance of the training activities** | Proposed activities are not realizable, they doesn’t fit the proposed methodology or they are not adapted to the Primary Education course indicated | Proposed activities are realizable, they are adapted to the Primary Education course indicated but they are not linked to the methodology proposed | Proposed activities are realizable, they are adapted to the Primary Education course and they are adequate for the methodology proposed | 0-2 |
| **Correction of evaluation procedures** | TU doesn’t meet the evaluation criteria and standards. The evaluation technic and instruments are not adequate, and it doesn’t propose the evaluation sessions | Evaluation criteria and standards are fixed. Most of the technics and instruments are adequate and it propose the evaluation sessions | Evaluation criteria and standards are fixed. Technics and evaluation instruments are all adequate and it explains correctly the evaluation sessions | 0-2 |
| **Content adequacy (PANEL OF EXPERTS)** | Contents are not adequate for the course proposed or they are not discussed | Contents are adequate for the course proposed but they are not discussed | Contents are adequate for the course proposed and they correctly discussed | 0-2 |
| **Tools & resources adequacy**  **(PANEL OF EXPERTS)** | TU uses a short variety of resources or they are not adequate for the proposed activities | TU uses a wide variety of resources but some of them are not adequate to the content or the proposed activities | TU uses a wide variety of resources, they are adequate to the theoretical frame, the contents and the proposed activities | 0-1 |
